# *QuickStats:* Percentage of Adults Who Met Federal Guidelines for Aerobic Physical Activity Through Leisure-Time Activity,* by Race/Ethnicity — National Health Interview Survey,^†^ 2008–2017

**DOI:** 10.15585/mmwr.mm6812a6

**Published:** 2019-03-29

**Figure:**
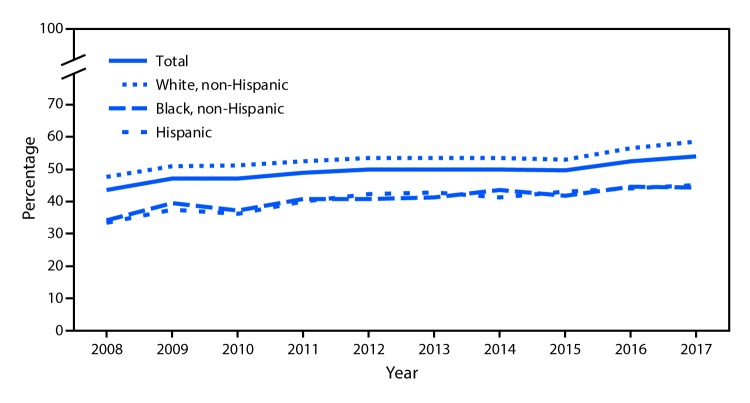
During 2008–2017, the percentage of adults aged ≥18 years who met federal guidelines for aerobic physical activity through leisure-time activity increased from 43.5% in 2008 to 54.1% in 2017. This pattern was seen in each race/ethnicity group shown, with an increase from 33.4% to 45.0% for Hispanic, 34.1% to 44.3% for non-Hispanic black, and 46.0% to 58.6% for non-Hispanic white adults. Throughout the period, non-Hispanic white adults were more likely to meet the guidelines through leisure-time activity than were non-Hispanic black and Hispanic adults.

**Source:** National Health Interview Survey, 2008–2017. https://www.cdc.gov/nchs/nhis/index.htm.

